# Validation of NEWS2 as a triage tool for ambulance transport requests for patients assessed by healthcare professionals

**DOI:** 10.1186/s12873-026-01626-4

**Published:** 2026-06-05

**Authors:** Ola Græsli, Thomas Christensen, Pieter Jelle Toussaint

**Affiliations:** 1 Division of Prehospital Services, Oslo University Hospital, Oslo, Norway; 2 Department for Prehospital Emergency Medicine, Institute for Nursing and Health Promotion, Oslo Metropolitan University, P.O. Box 4, St. Olavs plass, Oslo, NO-0130 Norway; 3 Norwegian University of Science and Technology, Trondheim, Norway

**Keywords:** Clinical assessment, Deterioration, Digitalization, EMCC, EMS, Monitoring, Mortality, NEWS2, Patient safety, Quality improvement

## Abstract

**Background:**

The National Early Warning Score (NEWS2) system, commonly used in hospitals, has shown potential to be an effective prehospital triage tool for ambulance transport requests. This study evaluates the application of NEWS2 for triaging patients in need of transport by ambulance, after assessment by a healthcare professional. The study aims to validate an electronic triage form developed at Oslo Emergency Medical Communication Center (EMCC), focusing on its correlation with patient outcomes and the impact of operator-adjusted emergency codes.

**Methods:**

A retrospective cross-sectional analysis was conducted on 36,865 ambulance transport cases documented at Oslo EMCC from December 2018 to December 2020. Data from the electronic triage form, EMCC’s journal records and the Norwegian Cause of Death Registry were linked and analyzed to assess the relationship between NEWS2 scores, emergency codes and patient mortality. Associations between NEWS2 score and mortality at 1, 7 and 30 days were assessed using univariable logistic regression analyses, alongside descriptive sensitivity and specificity analyses. Operator adjustments to system-recommended emergency codes were also evaluated for their influence on outcomes.

**Results:**

Increasing NEWS2 scores were significantly associated with increased mortality at 1, 7 and 30 days, with the strongest association observed for 1-day mortality. In total 40% of cases were upgraded by either the system (17%) or the operator (23%). System-initiated upgrades effectively identified critically ill patients, while operator-initiated adjustments showed minimal impact on mortality.

**Conclusions:**

The findings support the use of NEWS2 as a reliable tool for prehospital triage, aligning with in-hospital findings and supporting consistent patient assessments. While operator discretion plays a role in refining urgency, its impact on mortality was not always significant. The results emphasize the importance of accurate triage to optimize patient outcomes and highlight the need for ongoing evaluation and integration of NEWS2 into EMCC practices. Future research should explore additional outcome measures beyond mortality to assess the broader efficacy of NEWS2 in prehospital settings.

**Clinical trial number:**

Not applicable.

**Supplementary Information:**

The online version contains supplementary material available at 10.1186/s12873-026-01626-4.

## Introduction

In Norway, emergency medical responses are primarily managed by ambulances, responding to public calls for acute illness or injury, as well as for patient transfers requiring monitoring or urgent care [1]. The Emergency Medical Communication Centers (EMCC) coordinate these ambulance dispatches, determining the priority and allocation of available resources.

EMCCs receive all calls to the medical emergency number 113, alongside referrals from other health services, police, fire departments and healthcare professionals. Each call is triaged, employing the Norwegian Index for Emergency Medical Assistance (NIMN), a standardized, symptom-based tool that categorizes cases by urgency level [2]. While NIMN has ensured consistent triage for patients without prior medical assessment, cases involving patients already evaluated by healthcare professionals present challenges due to additional clinical data not covered by standard protocols. This highlights the need for enhanced decision-support tools to ensure equitable and accurate prioritization. All patients are triaged by the EMCC, but the importance of accurate prioritization increases during periods where mission demand exceeds available resources. Patients are generally triaged using NIMN, but when the patient has been assessed by healthcare professionals, there are additional data available and an opportunity for a more accurate triage. At the same time this might address the differences between patients assessed in person and patients assessed over the phone.

Missions in the EMCC in Oslo are triaged into four different emergency codes, based on type of incident and urgency level. The most critical conditions are categorized as “A” (priority 1), which means a potentially life-threatening or life-threatening condition. These missions should be dispatched as soon as possible and the ambulances will use lights and sirens. Conditions categorized as “H” (priority 2) are conditions that should be addressed as soon as possible, but not by lights and sirens and some waiting time is accepted if resource coverage is limited. This is to ensure there are resources available for high acuity missions. The emergency codes “V1” (priority 3) and “V2” (priority 4) are used for conditions that can wait and planned transports. “V1” is used for conditions that do not need an immediate response, but a timely response is still required. “V2” is used for planned transports, often between different hospitals and institutions. For these transports the patient’s condition is either considered stable, or the patient is monitored and appropriately managed at their current location.

Clinical decision support systems (CDSS) have demonstrated effectiveness in standardizing patient evaluations and supporting clinical decisions [3]. The National Early Warning Score (NEWS2), a widely recognized CDSS in hospitals, assesses vital parameters to guide triage and interventions [4]. NEWS2’s use has extended to prehospital settings, showing promising results [5]. However, there is limited research on its application for triaging patients assessed by healthcare professionals before ambulance arrival.

To address this, Oslo EMCC developed and implemented an electronic NEWS2-based ambulance request form in 2018, for cases involving patients assessed by healthcare professionals (see Supplementary Fig. 1). This form includes adaptations such as psychiatric considerations, the Pediatric Early Warning Score (PEWS) for pediatric cases and the quick Sequential Organ Failure Assessment (qSOFA) for suspected sepsis. Based on inputted information, it provides an emergency code recommendation, that the operators are able to adjust based on clinical judgment and input from the caller. For NEWS2 the baseline emergency code is given by the recommended NEWS2 categories [6]. This means that category 1 (NEWS2 score ≥ 7) corresponds to “A”, category 2 (NEWS2 5 to 6) corresponds to “H” and category 3 (NEWS2 score 0 to 4) corresponds to “V1”. The form was not designed for “V2” missions, as this emergency code is primarily used for planned transports. Consequently, the form itself does not generate a “V2” recommendation. However, operators may manually adjust a recommended “V1” emergency code to “V2” in cases considered appropriate for planned transport. In addition to the operator adjustment, the system also has some built in minimum emergency codes, for special conditions like a heart condition with ongoing chest pain and high NEWS2 in one parameter. The form is provided in a separate window and is not integrated with other software in the EMCC. Operators open it in a web browser and fill out the requested information. This provides the operator with a summary of the information, which is then copied into the Computer Aided Dispatch (CAD) system, where information about locations and patients are added. In that process the operator decides if they follow the emergency code provided by the form summary or adjust it. There are not defined any criteria for emergency code adjustment, other than the built in adjustments.

When someone calls the EMCC with the need for an ambulance, it is treated either as an emergency or a transport request. The NEWS2-based electronic request form evaluated in this study was used specifically for ambulance transport requests involving patients already assessed by healthcare professionals. For emergencies they are assessed by healthcare professionals and based on the phone interview the call taker categorizes it with an urgency level according to NIMN. These call takers are mostly nurses and paramedics, with extensive training in medical emergencies over the phone. NIMN provides some support in regards of what to ask for and rule out, but it is up to the call taker how the interview is organized, as long as all more acute symptoms in the selected chapter in NIMN are excluded. Transport requests are handled by the same call takers as medical emergencies, but also dispatchers and other roles in the EMCC can answer those. All operators have training in handling transport requests. They are handled by prompting the caller for information to fill out the ambulance request form. Based on the results from that the operator and the caller have the option to adjust the suggested emergency code. This ensures that all patients and their missions are triaged within the defined emergency codes. All call takers have the option to transfer to or consult call takers trained for handling medical emergencies, if they are not sure about the urgency of a transport request.

This study aims to validate the electronic ambulance request form, by assessing the correlation between NEWS2-based triage recommendations, emergency code adjustments by operators and patient mortality. Mortality has frequently been used as an outcome measure in studies evaluating NEWS2, allowing comparison across hospital and prehospital settings despite the limited research on prehospital telephone triage using NEWS2 [6, 7]. Comparing the findings with existing hospital and prehospital data will provide insights into the form’s utility and guide future applications of NEWS2 in prehospital triage.

## Method

This retrospective cross-sectional study validates the suitability of the NEWS2 scoring system for triaging ambulance transport requests involving patients already assessed by healthcare professionals. The study compared NEWS2-based triage levels and emergency code allocation with patient mortality as the primary outcome.

### Data sources

Data were extracted from two main systems: Acute Medical Information System (AMIS), the EMCC journal records and documentation system and a custom database containing structured data from the electronic ambulance transport request form. These sources were linked via a unique “request ID” used by EMCC operators. Mortality data were sourced from the Norwegian Cause of Death Registry, managed by the Norwegian Institute of Public Health, which provided information on cause and date of death [8]. This linkage was achieved using patients’ personal identification numbers, ensuring comprehensive data across different hospitals and systems.

### Sample selection

The study included ambulance requests from December 1st, 2018, to December 31st, 2020, to align with the introduction and subsequent use of the electronic ambulance request form. Planned transports, such as home transports and next day transfers are exempted from the request form and were not included in the study.

#### Inclusion criteria

Only cases with a valid NEWS2 score, valid personal identifier and successful linkage between databases were included. Exclusions were made for psychiatric cases, where NEWS2 was not applicable, forced admissions and patients under 18 years of age, who were assessed using PEWS instead. In addition, incidents categorized through the predefined acute section of the form, where predefined life-threatening clinical conditions triggered immediate dispatch independent of NEWS2 scoring, were excluded because NEWS2-based triage was not operationally relevant in these situations.

**Fig. 1:**
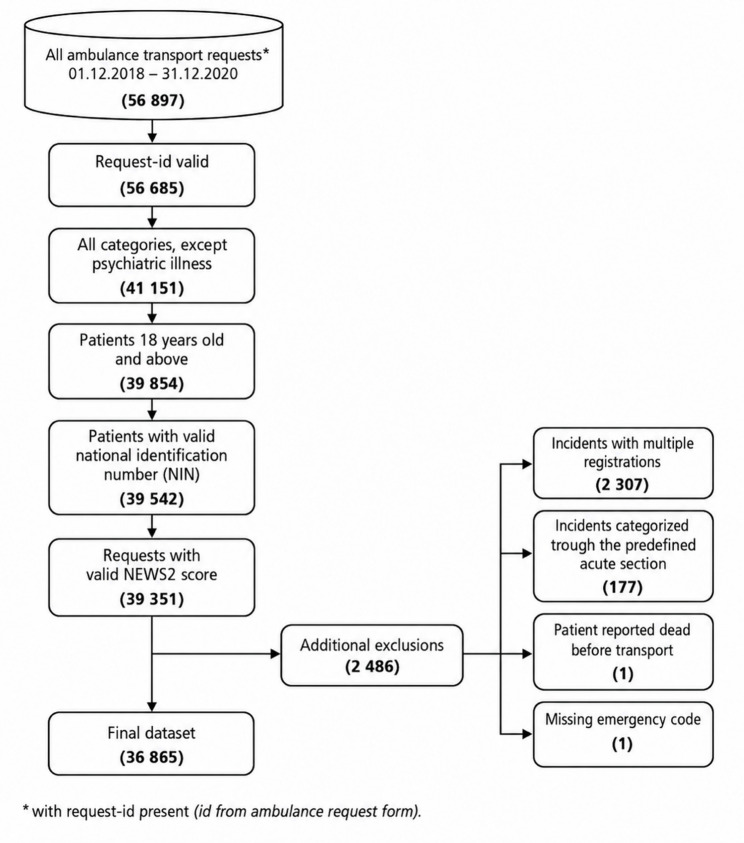
Flowchart of study inclusion and exclusion

During the study period, individual patients could have multiple ambulance transport requests. Each transport request was treated as a separate incident in the analysis. Duplicate records identified during data cleaning were removed. The inclusion process is shown in Fig. 1.

### Data integration

Data integration involved merging the datasets using the “request ID” and patient identification numbers. Cases without valid identifiers or successful linkage between datasets were excluded from analysis, as described in Fig. 1. After linkage, identifying information was deleted to ensure anonymity. This process resulted in a dataset stripped of identifying details, including modified timestamps to maintain patient privacy, while retaining the essential analytical parameters required for the study.

### Data analysis

Given the broad range of conditions covered by the ambulance request form, mortality was chosen as a primary parameter due to its relatively unbiased representation of patient severity and triage effectiveness, even though it may not fully capture delayed treatment impacts. The analyses examined correlations between recorded NEWS2 scores and mortality rates, considering time intervals of 1-, 7- and 30-day post-event. This stratification aimed to gauge NEWS2’s predictive precision over varying periods, with a primary focus on 30-day mortality for broader insight.

The NEWS2 scores from the electronic ambulance request form were grouped into three operational categories based on the NEWS2 escalation thresholds described by the Royal College of Physicians [6]. Category 1 (NEWS2 score ≥ 7) corresponded to emergency code “A”, category 2 (NEWS2 score 5–6) corresponded to “H” and category 3 (NEWS2 score 0–4) corresponded to “V1”. These categories reflected the operational emergency code recommendations implemented in the electronic request form. The categories were used descriptively to assess mortality gradients across operational emergency code thresholds.

Univariable logistic regression analyses were performed to assess the association between NEWS2 score and mortality at 1, 7 and 30 days. Mortality outcomes were treated as binary dependent variables and NEWS2 score as a continuous independent variable. Odds ratios (ORs) with 95% confidence intervals (CIs) were calculated for each 1-point increase in NEWS2 score.

Analyses were conducted using Python, leveraging Pandas for data manipulation, and PyPlot and Seaborn for visualization. The dataset underwent data cleaning to identify inconsistent or incomplete records, including negative time-to-death entries and incidents with multiple registrations, as described in Fig. 1.

Additional analyses examined the relationship between emergency code allocation, operator overrides, mortality and time to hospital arrival. Operator-adjusted emergency codes were compared with the standard system recommendations to assess how manual adjustments corresponded to patient outcomes.

### Ethical considerations

The study adhered to current ethical guidelines and was approved by the Regional Committees for Medical and Health Research Ethics (REK) (nr: 230374), the Norwegian Center for Research Data (NSD) (nr: 906354) and the privacy units at Norwegian University of Science and Technology (NTNU) and Oslo University Hospital (OUH). Sensitive data were anonymized post-integration and approval for waiving patient notification was granted by REK due to the study’s retrospective design and large sample size.

## Results

The dataset included 36,865 ambulance transport requests. Table 1 summarizes mortality rates categorized by NEWS2 scores and urgency levels. Mortality was assessed at 1 day, 7 days and 30 days post-incident to evaluate how well NEWS2 correlates with patient outcomes. The median age of included patients was 67.3 years, with a nearly equal distribution between genders.

**Table 1:** Baseline characteristics by cumulative mortality group

	Incidents, *n* (%)	Age, median (IQR)	Men*, *n* (%)	NEWS2 0–4, category 3	NEWS2 5–6, category 2	NEWS2 > = 7, category 1
No 30-day mortality	34 171 (92.7%)	71 (53–82)	16 973 (49.7%)	26 403 (77.3%)	4 339 (12.7%)	3 429 (10.0%)
1-day mortality	197 (0.5%)	83 (73–88)	99 (50.3%)	75 (38.1%)	45 (22.8%)	77 (39.1%)
7-day mortality	958 (2.6%)	82 (74–89)	494 (51.6%)	456 (47.6%)	208 (21.7%)	294 (30.7%)
30-day mortality	2 694 (7.3%)	82 (74–89)	1 423 (52.8%)	1 553 (57.6%)	510 (18.9%)	631 (23.4%)
Total	36 865 (100.0%)	72 (54–83)	18 396 (49.9%)	27 956 (75.8%)	4 849 (13.2%)	4 060 (11.0%)

*Three patients were registered without gender. These are excluded in analysis based on gender

Men and NEWS2 categories are reported as n (% within the corresponding mortality group). Mortality outcomes were cumulative: patients deceased within 1 day were also included in the 7-day and 30-day mortality groups, and patients deceased within 7 days were also included in the 30-day mortality group

**Fig. 2:**
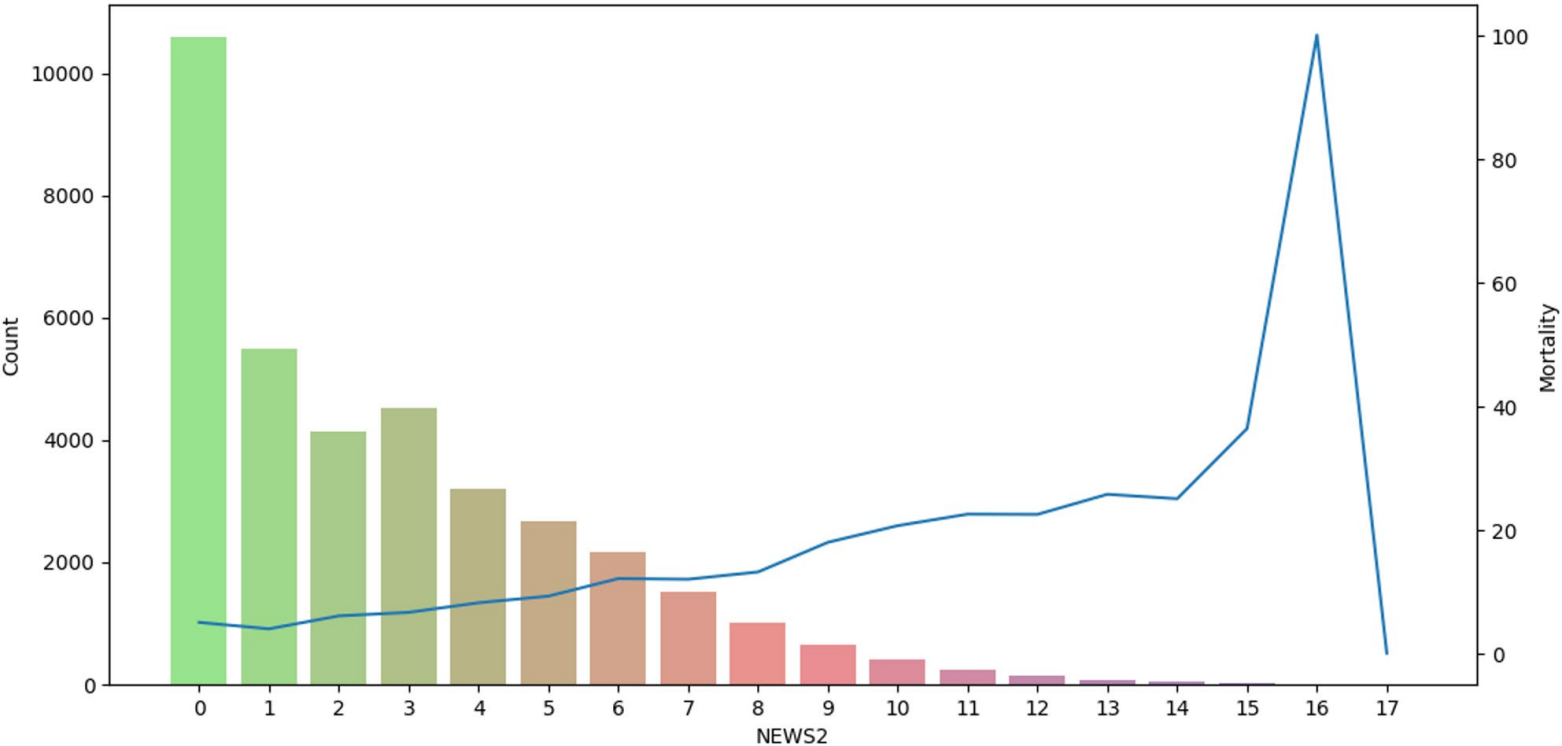
30-day mortality (%) by NEWS2 score (line) and number of incidents (bars). The highest NEWS2 scores represented relatively few observations

The analysis confirmed a strong association between higher NEWS2 scores and higher 30-day mortality (Fig. 2). Mortality rates rose progressively with higher NEWS2 categories, indicating that the operational NEWS2 thresholds effectively stratified patients by mortality risk. Mortality within 30-day for patients in NEWS2 category 3 (score 0–4) was 5.6% and increased to 15.6% in category 1 (score ≥ 7). Increasing NEWS2 scores were significantly associated with increased mortality at 1, 7 and 30 days. The strongest association was observed for 1-day mortality, while the association gradually reduced over longer follow-up periods (Table 2). Each 1-point increase in NEWS2 was associated with a 30% increase in the odds of 1-day mortality.

The highest observed NEWS2 scores represented relatively few incidents. NEWS2 score 16 included four incidents, while NEWS2 score 17 included one incident, and the corresponding mortality estimates should therefore be interpreted cautiously.

**Table 2:** Association between NEWS2 score and mortality

Outcome	*n*	Deaths	OR per 1-point NEWS2 increase	95% CI	*p*-value
One day	36 865	197	1.30	1.26–1.36	< 0.001
7 days	36 865	958	1.24	1.21–1.26	< 0.001
30 days	36 865	2 695	1.18	1.16–1.19	< 0.001

### Sensitivity and specificity analysis

Table 3 presents sensitivity and specificity analyses for NEWS2 scores ≥ 5 (categories 1 and 2) and ≥ 7 (category 1). For 1-day mortality, the specificity was high (76% for scores ≥ 5 and 89.1% for scores ≥ 7), although sensitivity was moderate (61.9% and 39.1%, respectively). The high negative predictive value (99.7% and 99.6%) indicated that patients with lower scores had a low probability of mortality. These results were consistent over 30-day mortality assessments (Table 3), with slightly reduced sensitivity and similar specificity.

**Table 3:** Sensitivity, specificity and accuracy

	1-day mortality		7-day mortality		30-day mortality	
**NEWS2**	≥ 5^**^	≥ 7^*^	≥ 5^**^	≥ 7^*^	≥ 5 ^**^	≥ 7^*^
**Sensitivity**	61.9%	39.1%	52.4%	30.7%	42.4%	23.4%
**Specificity**	76.0%	89.1%	76.6%	89.5%	77.3%	90.0%
**Positive predictive value**	1.4%	1.9%	5.6%	7.2%	12.8%	15.5%
**Negative predictive value**	99.7%	99.6%	98.4%	98.0%	94.4%	93.7%
**False positive rate**	24.0%	10.9%	23.4%	10.5%	22.7%	10.0%
**False negative rate**	38.1%	60.9%	47.6%	69.3%	57.6%	76.6%
**False discovery rate**	98.6%	98.1%	94.4%	92.8%	87.2%	84.5%
**Accuracy**	76.0%	88.9%	76.0%	88.0%	74.7%	85.1%

* Categroy 1

** Category 2

### Mortality by emergency codes

**Fig. 3:**
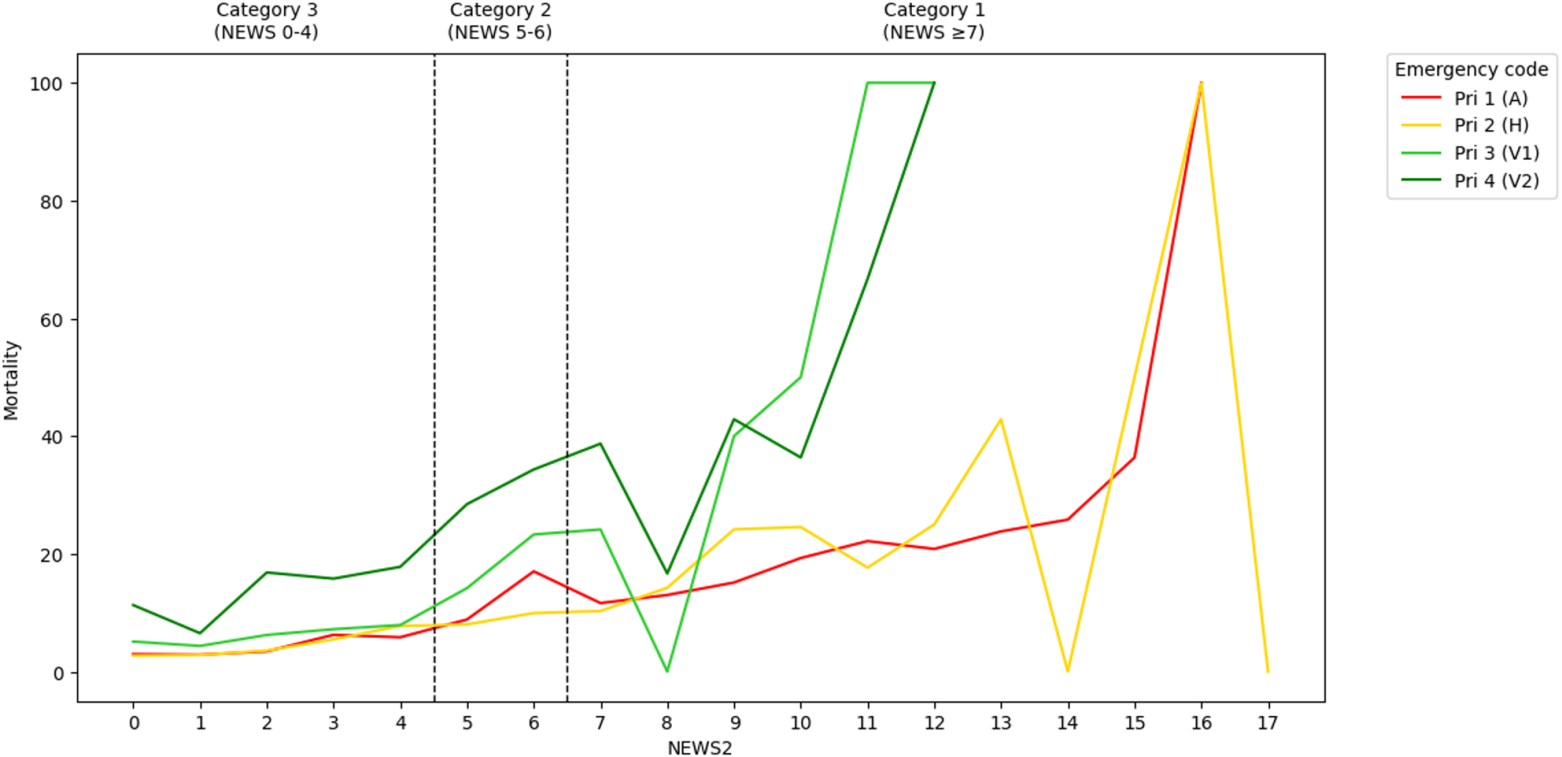
Mortality by NEWS2 score, split by emergency code (30-day mortality)

Examining 30-day mortality across emergency codes (Fig. 3), patients triaged with lower urgency (V1 and V2) showed some mortality within NEWS2 category 3 (0–4). Some of these patients may include transports to and from palliative care or other patients that are very sick but do not necessarily benefit more from staying at the hospital. For NEWS2 category 2 (5–6), there was notable mortality in patients triaged at V1, suggesting that while downgraded urgency was justified by clinical discretion, it could still pose risks. In category 1 (≥ 7), mortality was consistently high across emergency codes. The results do not account for possible effects of different emergency codes on mortality, in regards of priority before or after other incidents and time to hospital.

### Mortality by point of origin

**Fig. 4:**
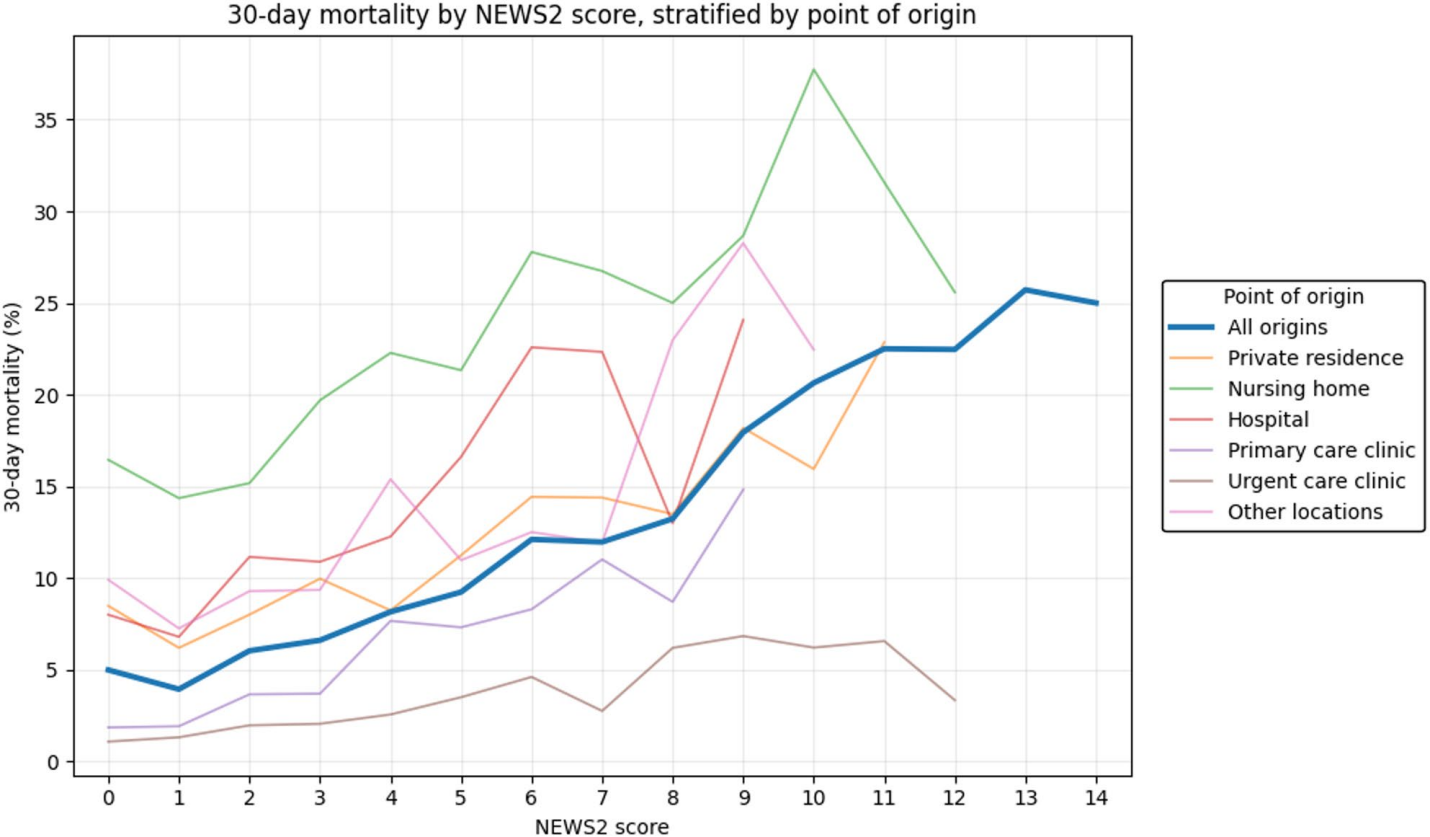
30-day mortality by NEWS2 score stratified by point of origin. Observed NEWS2 scores in this analysis ranged from 0 to 14

Stratified analyses by point of origin showed that higher NEWS2 scores were associated with increased 30-day mortality across all settings, with similar overall trends despite differences in absolute risk (Fig. 4). Similar patterns were observed for 1- and 7-day mortality.

### Time to hospital arrival

**Fig. 5:**
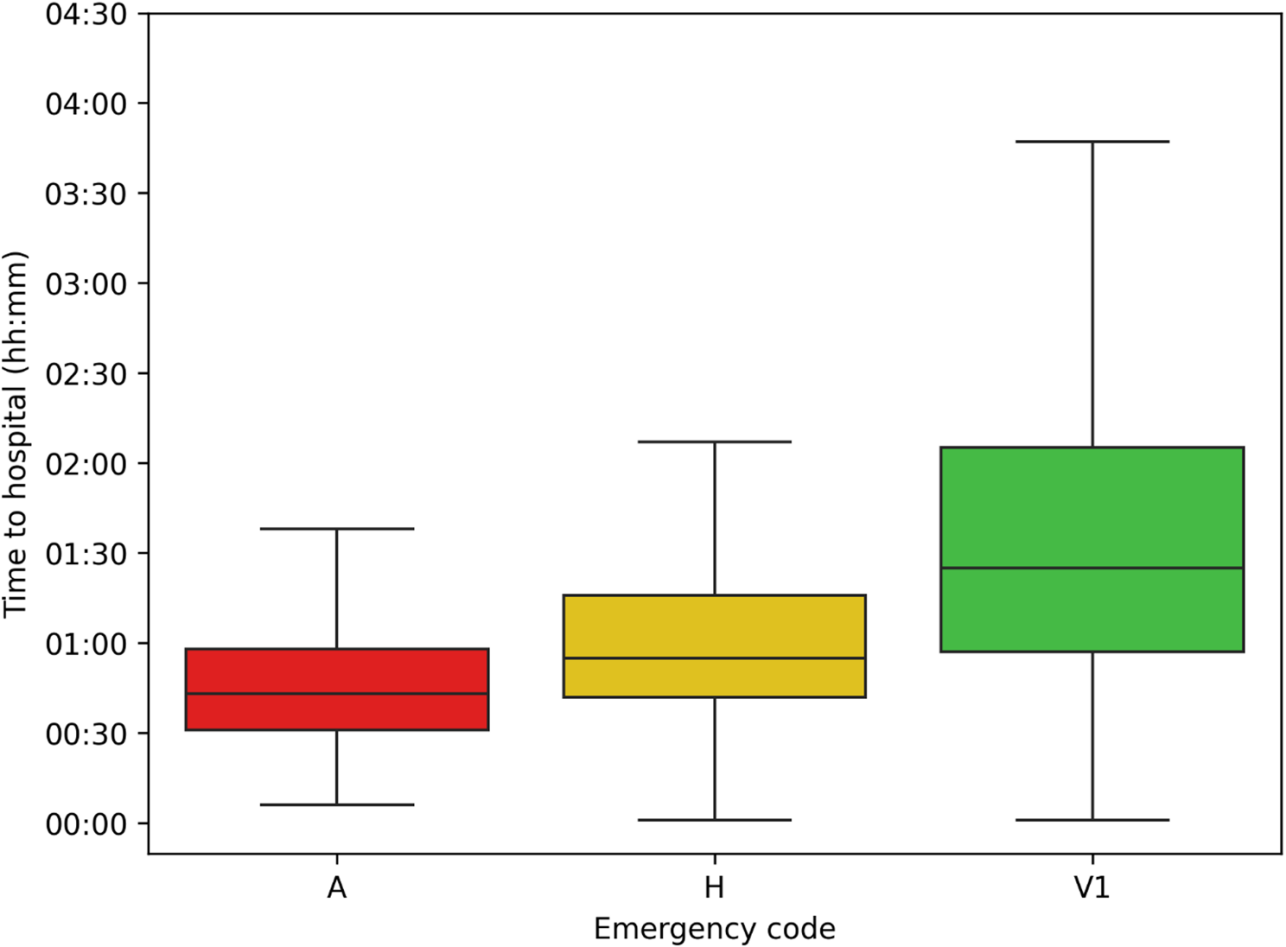
Time from request to hospital arrival by emergency code (excluding > 5 h)

As emergency code allocation directly affects operational prioritization, time from first request to hospital arrival was analyzed across emergency codes (Fig. 5, Table 4). Median time to hospital arrival was shortest for emergency code A (43 min), followed by H (55 min) and V1 (85 min). Figure 5 illustrates the increasing variability and delay associated with lower-priority emergency codes.

**Table 4:** Time to hospital per emergency code in minutes

Emergency Code	*n*	Mean	SD	25th percentile	Median (50th percentile)	75th percentile
A	4,553	47.4	24.3	31	43	58
H	15,330	62.6	31.9	42	55	76
V1	11,744	97.4	54.2	57	85	125

Excluding 465 incidents > 5 h

### Operator-adjusted emergency codes

**Fig. 6:**
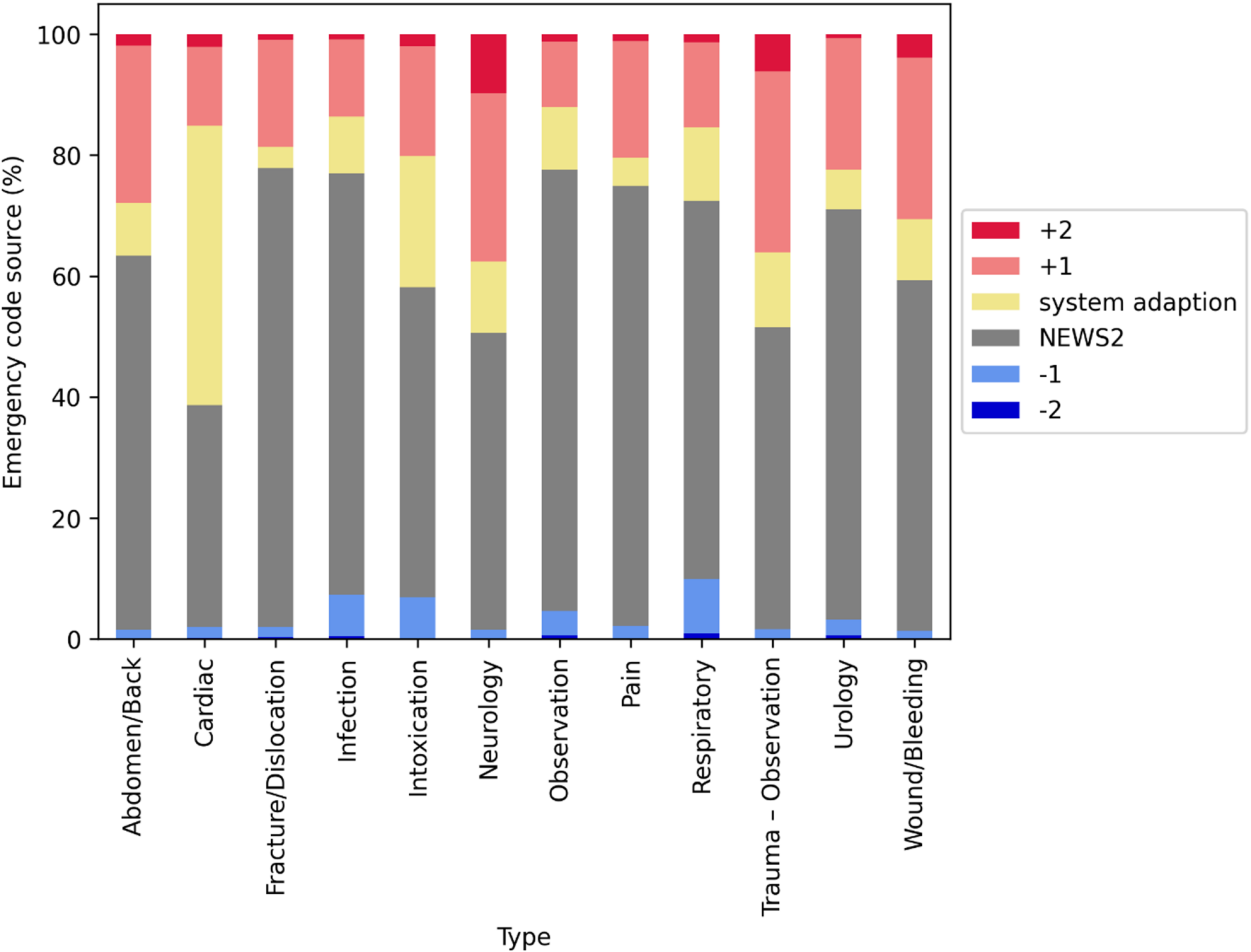
Percentage of overrides per incident type

The adjustment of emergency codes by operators is a notable aspect of this study, as shown in Fig. 6. This figure highlights the proportion of cases within various incident types where emergency codes were adjusted. Adjustments were categorized based on whether the urgency was increased (+ 1 or + 2) or decreased (-1 or -2) by the operator. Additionally, a “system adaptation” category was included for instances where the system itself upgraded the urgency due to specific patient criteria, such as ongoing chest pain, a NEWS2 sub-score of 3 in one category, or a positive qSOFA score. System-triggered upgrades typically moved cases from “routine” (category 3) to “urgent” (category 2). In total, 23% of the included incidents were adjusted by the user and 17% were adjusted by the system. Looking at upgrades, all of the system adjustments were upgrades, while 20% of the incidents were upgraded by the user.

Analysis revealed that certain incident types were more frequently adjusted, either by the system or the operator. The most common operator adjustment was an upgrade by one emergency code.

**Fig. 7:**
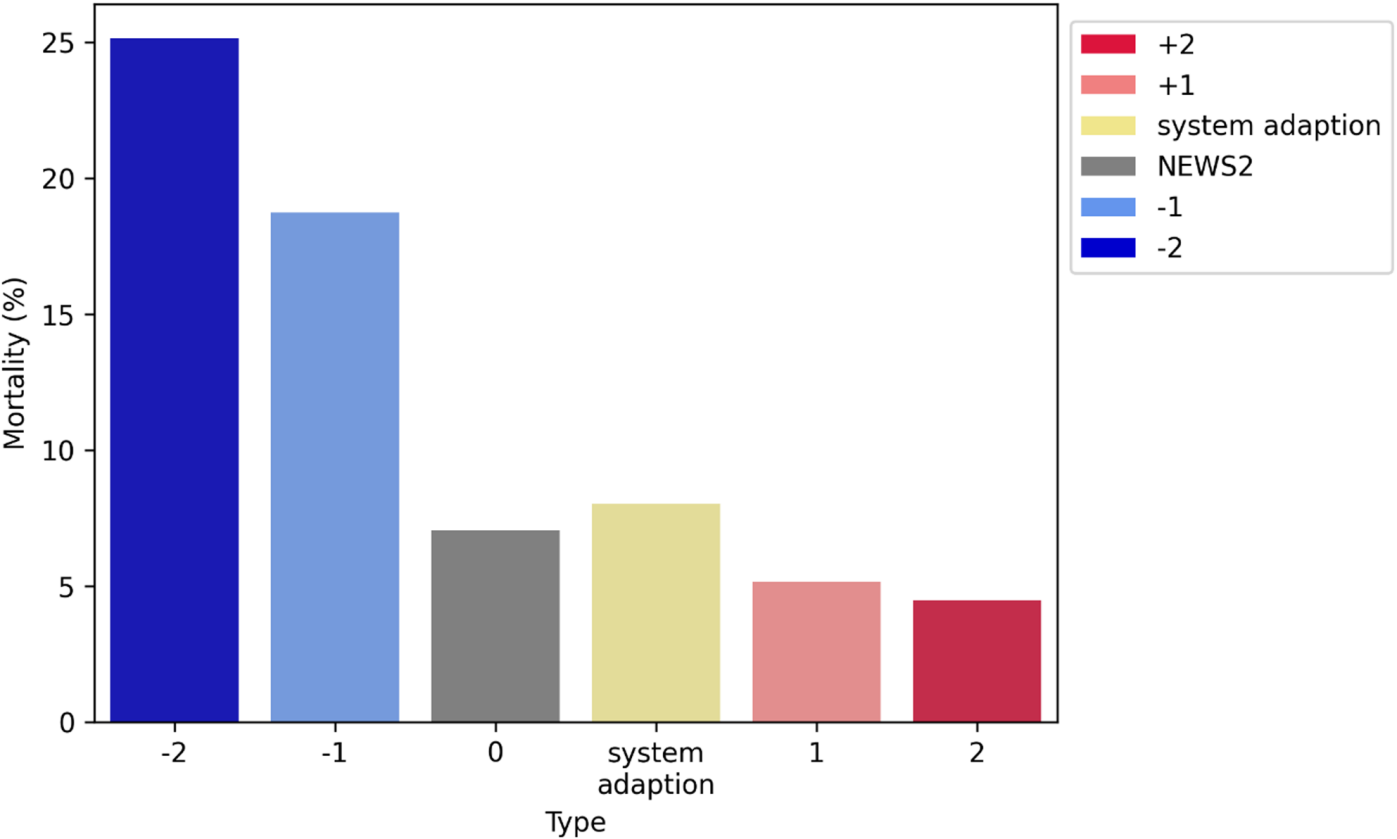
Mortality per override type (30-day mortality)

Figure 7 presents 30-day mortality rates across different incident types, comparing cases where emergency codes were adjusted with cases without adjustment. Mortality was generally higher among patients whose urgency was reduced. Cases with system-initiated urgency upgrades also demonstrated higher mortality in several incident categories. However, mortality patterns varied between incident types, including cardiac, intoxication and neurological incidents.

## Discussion

This study has provided substantial evidence supporting the use of the NEWS2 scoring system for triaging ambulance transport requests at EMCCs. The findings indicate that NEWS2 effectively correlates with patient mortality, making it a reliable tool for assessing the urgency of cases. The study’s results align with prior research on the predictive validity of NEWS2 both in hospital and prehospital settings, reinforcing its value as a consistent triage metric [6, 7, 9–11].

These findings are important for patient triage and operational prioritization in the EMCC. As the Emergency Medical Services (EMS) resources used for ambulance transport requests are the same as those who responds to emergency calls from the public, it is important to have triage systems that ensures that the patients get as correct triage as possible by the EMCC. Based on the findings NEWS2 with some modifications can be a good tool to triage requests for ambulance transports when the patient has already been assessed by a health care professional. One example can be a patient with ongoing chest pain. If that patient called the EMCC, the triage will often be “A” (priority 1). Then if the same patient has been assessed by a doctor and is clinically stable, the triage of the transport to hospital would be “H” (priority 2), if immediate intervention is not deemed necessary. This difference can play an important role when demand is high and resources are limited.

The analysis showed a clear relationship between increasing NEWS2 scores and increased mortality across all follow-up periods. The association was strongest for 1-day mortality and gradually reduced over longer follow-up periods, which is expected as NEWS2 primarily reflects acute physiological disturbance. These findings support the use of NEWS2 as a clinically relevant decision-support tool during telephone triage of ambulance transport requests for patients already assessed by healthcare professionals. The predefined NEWS2 categories also demonstrated progressively increasing mortality rates, suggesting that the operational thresholds used to assign emergency codes corresponded to clinically meaningful differences in patient risk.

Mortality remained elevated across emergency codes in NEWS2 category 1 (≥ 7), underscoring the importance of careful assessment when considering lower-priority emergency code allocation in patients with high NEWS2 scores. At the same time, some patients within these groups may have had advanced illness or palliative treatment goals, highlighting the complexity of triage decisions in this population. To conclude one need to look more into the history of each patient. A limitation of the analysis is the decided emergency codes for different NEWS2 scores. The emergency code is a contributor to prioritization of the missions and will therefor affect average time to hospital. This might affect the mortality of that NEWS2 score. To mitigate this the categories used was based on the initial recommendations in NEWS2, which seems to be supported by research from inside hospitals. Limited prehospital studies, which does not look at prehospital emergency codes used in Norway makes this mapping of categories an uncertainty. The findings suggest that the prognostic performance of NEWS2 is robust across different points of origin, although contextual differences in baseline risk and care may influence absolute outcomes.

Stratified analyses by point of origin demonstrated similar overall mortality trends across different healthcare settings, with increasing NEWS2 scores associated with higher mortality regardless of origin. Although absolute mortality differed between settings, likely reflecting differences in baseline patient populations and levels of ongoing care, the consistent mortality gradients support the robustness of NEWS2-based triage across different clinical contexts.

The sensitivity and specificity analyses further support the operational utility of NEWS2 in this setting. The high negative predictive values observed for lower NEWS2 thresholds suggest that patients with low NEWS2 scores had a low probability of short-term mortality, supporting the role of NEWS2 as a tool for identifying lower-risk patients during ambulance transport triage. In contrast, the relatively low positive predictive values were expected given the overall low mortality prevalence in the study population and reflect the intended use of NEWS2 as a triage and risk stratification tool rather than a definitive predictor of mortality. These findings are consistent with previous studies evaluating NEWS2 in both hospital and prehospital settings [6, 7, 9–11].

Although this study was not designed to directly evaluate whether NEWS2 improves triage performance compared to existing practice, the findings suggest that NEWS2 may provide clinically useful decision support in ambulance transport triage. The observed mortality gradients, combined with high negative predictive values for lower NEWS2 thresholds, indicate that the system may help identify lower-risk patients while supporting consistent prioritization across operators. In addition to risk stratification, the structured collection of physiological parameters may contribute to more standardized communication between healthcare professionals and EMCC operators. At the same time, the use of NEWS2 requires additional information gathering and documentation and its operational benefit must therefore be balanced against increased workload and workflow complexity.

The differences in time to hospital arrival between emergency codes illustrate the operational consequences of emergency code allocation. Patients assigned the highest urgency (A) were, as expected, transported more rapidly than those assigned lower priority emergency codes, while the greatest variability in waiting time was observed in V1 missions. Although the median difference between A and H was relatively small, this distinction may still be important for patients requiring time-critical interventions. At the same time, the relatively limited difference between these categories may support the use of H for clinically stable patients, helping preserve immediate response for the most critical incidents. Since the predefined NEWS2 categories formed the basis for emergency code recommendations, these findings demonstrate how NEWS2-based triage directly influences operational prioritization within the EMS system. The greater delays and variability observed in lower-priority categories also highlight the importance of accurate triage, as both overtriage and undertriage may have operational and clinical consequences.

Regular waiting times of up to four hours for non-urgent patients (V1) highlight the importance of accurate triage to prioritize patients needing quicker response. At the same time, one can argue that the difference between A and H might not be critical for most patients. This can indicate that differentiating between A and H is not as critical, if patients in need of time critical interventions are identified.

Operator adjustments to emergency codes highlight the complexity of clinical decision making. Approximately 30% of cases were upgraded by either the system or the operator (Fig. 6), underscoring the role of clinical judgment in refining automated recommendations. Interestingly, system-driven urgency upgrades effectively captured critically ill patients, supporting the systems utility. However, operator-initiated urgency adjustments did not consistently show a significant impact on mortality (Fig. 7), suggesting that while these modifications may be clinically justified, they might not always influence outcomes in terms of mortality. This was particularly evident for cases categorized as cardiac, intoxication and neurological events, where mortality rates among upgraded cases were similar to those without urgency changes. In these situations, urgency adjustments may have limited influence on mortality, with other clinical factors likely contributing more substantially to outcomes. The results also highlight the need for caution with downgrading urgency, as mortality for downgraded patients varied, underscoring the complexity and risk associated with these decisions. This emphasizes the importance of balancing system recommendations with professional judgment to optimize patient outcomes. It is also important to notice that some of these cases might include severely ill patients, who would not have benefited from higher urgency and therefor were correctly downgraded. This finding warrants further exploration to assess the necessity and effectiveness of manual adjustments.

The implementation of a NEWS2-based request form may increase the amount of structured information collected from the requesting healthcare professional. However, because NEWS2 is already widely used within the healthcare system and is based on routinely assessed physiological parameters, this additional information collection may be operationally feasible in many settings. The observed association between NEWS2 and mortality, combined with the operational differences between emergency code categories, suggests that structured NEWS2-based triage may provide clinically relevant decision support during ambulance transport requests. At the same time, the balance between improved decision support and increased workflow demands should be considered in future implementation and evaluation studies.

## Limitations

Despite these promising findings, limitations include potential data inaccuracies due to the manual integration of datasets and retrospective nature of the study. Although quality controls and data validation measures were in place, the absence of a control group limits the ability to assess changes over time. Additionally, mortality as an outcome measure does not capture other significant patient impacts, such as quality of life or non-fatal complications, suggesting that future studies should incorporate broader metrics. The results do not account for the level of care the patient is under while waiting for transport, which might affect mortality. Operator adjusted cases might impact some of the results, as the measured outcome is based on the final emergency code and not the suggested emergency code from the system. Some of the included calls might be intensive care transports. These might have different needs and for example depend more on specialist personnel than emergency code and time to hospital. Therefor mortality based on emergency code and NEWS2 category, might not reflect patient need and be a good tool to measure mortality. Most of these will have manual adjustment, making their emergency code reflect actual patient needs. In the future qualitative insights could provide context regarding user experiences with the form and shed light to the operator adjusted cases.

The study intentionally excluded incidents categorized through the predefined acute section of the electronic request form, where predefined urgent clinical conditions triggered immediate dispatch independent of NEWS2 scoring. These cases were excluded because the urgency was primarily determined by the clinical condition itself rather than physiological risk stratification using NEWS2. The request form was intended for ambulance transport requests involving patients already assessed by healthcare professionals. However, the retrospective dataset did not allow assessment of how consistently the form was used in all eligible cases and some selection bias related to form utilization cannot be excluded.

## Conclusion

Use of the NEWS2-based electronic ambulance request form at Oslo EMCCs has proven to be effective for prehospital triage of patients requiring transport to a higher level of care, correlating well with patient mortality and aligning with established research. The system’s integration has facilitated consistent triage practices across operators, supporting patient safety and streamlined communication between prehospital and in-hospital teams. Nonetheless, the potential for overtriage and the nuanced impact of operator adjustments underscore the need for continuous assessment and refinement. Further studies should investigate the relationship between NEWS2 scores and critical interventions to provide a more comprehensive understanding of patient outcomes beyond mortality.

## Electronic supplementary material

Below is the link to the electronic supplementary material.

Supplementary Material 1

## Data Availability

Data for this study is available on Oslo University Hospital secure research server and is available through the corresponding author on reasonable request.

## Abbreviations

113: Norway’s Medical Emergency Number
AMIS: Acute Medical Information System
CDSS: Clinical Decision Support System
EMCC: Emergency Medical Communication Centre
EMS: Emergency Medical Services
NEWS2: The National Early Warning Score 2
