# Supplementary material for: Validation of NEWS2 as a triage tool for ambulance transport requests for patients assessed by healthcare professionals
Source: BMC Emerg Med. 2026 Jun 5;26:218. doi: 10.1186/s12873-026-01626-4 (PMC13465502; doi:10.1186/s12873-026-01626-4)
Supplement: Supplementary file 1 [file 12873_2026_1626_MOESM1_ESM.pdf]

Triage schema for ambulance transport requests

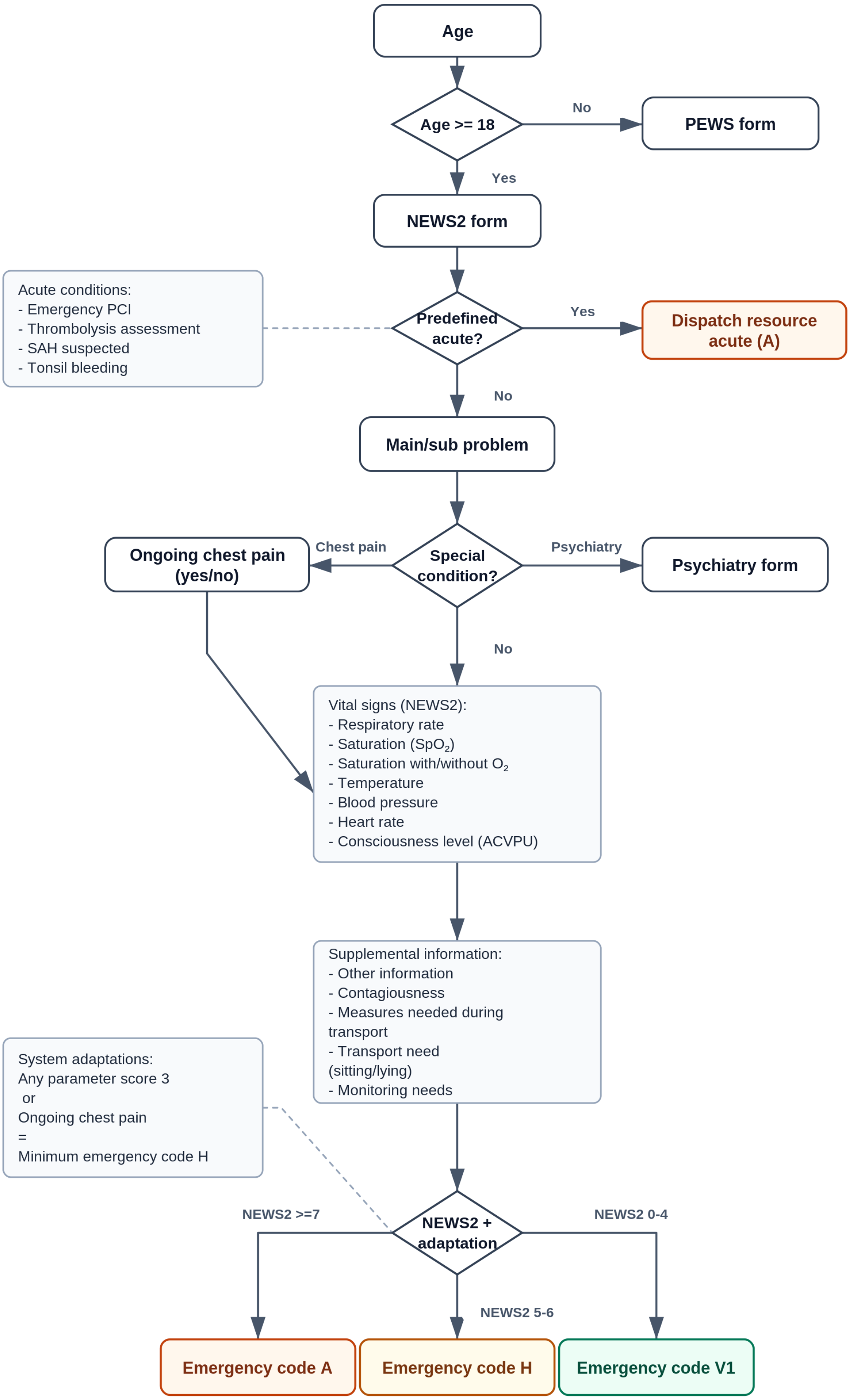

The figure describes a locally developed triage workflow used at EMCC Oslo, Prehospital Clinic, Oslo University Hospital. External implementation should only occur following local clinical and organizational approval. Reproduction, adaptation or operational use of the workflow should be clarified with EMCC Oslo.
